# Temporomandibular joint involvement in patients with Juvenile Idiopathic Arthritis: comparison of ultrasonography and magnetic resonance imaging in assessing the periarticular space width

**DOI:** 10.1007/s11282-023-00696-5

**Published:** 2023-07-03

**Authors:** Ingrid Tonni, Giulia Fossati, Maria Luisa Garo, Maria Grazia Piancino, Marco Cattalini, Luca Visconti, Andrea Borghesi

**Affiliations:** 1 Department of Medical and Surgical Specialties, Radiological Sciences and Public Health, Dental School, University of Brescia, P.za Spedali Civili 1, 25123 Brescia, Italy; 2 Department of Surgical Sciences, C.I.R. Dental School, Orthodontic Division, Dental School, University of Turin-Italy, Via Nizza 230, 10126 Turin, Italy; 3 Pediatrics Clinic, University of Brescia and ASST Spedali Civili of Brescia, Brescia, Italy; 4 Operational Unit 2nd Diagnostic Radiology, University of Brescia and ASST Spedali Civili of Brescia, Brescia, Italy

**Keywords:** Children, Juvenile Idiopathic Arthritis, Lateral Periarticular Width, Temporomandibular Joint, Ultrasound

## Abstract

**Objectives:**

This study aimed to compare the performance of Ultrasonography (US) and Magnetic Resonance Imaging (MRI) in assessing the Lateral Periarticular Space (LPAS) of Temporomandibular Joints (TMJs) in patients with Juvenile Idiopathic Arthritis (JIA).

**Methods:**

The LPAS width was evaluated in two different patient groups. In the JIA group, including 29 children (13 ± 2.8 years) with JIA, the LPAS width was measured with both MRI and US. In the healthy group, including 28 healthy children (12.6 ± 2.5 years), the LPAS width was measured only with US. Comparisons of LPAS width based on patient groups and TMJ contrast enhancement in MRI were evaluated by applying the Mann–Whitney *U* test. Correlation and agreement between MRI and US measurements in JIA group were tested using Spearman rank correlation and Bland–Altman method.

**Results:**

The LPAS width was significantly greater in the JIA group than in the healthy group. In the JIA group, the LPAS width was significantly greater in TMJs with moderate/severe enhancement than those with mild enhancement. A positive significant correlation between MRI and US measurements of LPAS width was found in the JIA group. In the same group, Bland–Altman method showed a good level of agreement between MRI and US measurements.

**Conclusion:**

Although, US cannot replace MRI in the evaluation of TMJ in patients with JIA, US could be used as a supplementary imaging method to MRI in assessing the TMJ disease.

## Introduction

Temporomandibular Joints (TMJs) are involved in 17–87% of patients affected by Juvenile Idiopathic Arthritis (JIA) depending on subtype, diagnostic criteria used, and ethnicity [1–8].

Joint involvement is characterized by episodes of active inflammatory arthritis leading to chronically progressive joint destruction. Further, JIA occurs mainly in the developmental age, and the presence of the disease at a temporomandibular level may severely compromise mandibular growth [9–11].

For this reason, an early diagnosis of TMJ involvement and a continuous follow-up are needed to reduce the TMJ damage and improve the quality of life in patients with JIA. This approach has the aim of monitoring the progress of the TMJ disease and modulating the therapeutic plan over time.

In active TMJ arthritis, pain is a rare symptom and very often children present with normal clinical examination findings [8, 12].

Currently, the heterogeneity of the published studies did not allow to find any clinical outcome measure that can be considered as predictor of TMJ involvement [13].

Clinical assessment is often insufficient for an early diagnosis of the TMJ involvement [14, 15]. Therefore, a standardized clinical examination protocol should be employed and studied in relation to the contrast-enhanced magnetic resonance imaging (CE-MRI) findings [16].

CE-MRI remains the gold standard for the evaluation of temporomandibular disease (TMD), given its capability to recognize the signs of the active (bone marrow edema, joint effusion, synovial thickening, joint enhancement) and chronic (bone erosion, condylar changes, and abnormalities of the articular disc) phases of the JIA at the TMJ level [17–20]. However, CE-MRI is costly, psychosocially burdensome, and not always practicable in young patients [10, 21]. MRI of the TMJ is a relatively long examination with an acquisition time of 30–45 min, requires intravenous injection of contrast medium, and sometimes sedation may be necessary [10].

Although MRI provides the best TMJ analysis due to its high-contrast resolution, ultrasonography (US) can provide complementary information on both condylar morphology and periarticular soft tissue. Therefore, US could be used to assess the TMJ involvement in JIA patients. However, its role in diagnosing arthritis of the TMJ is unclear. Some studies showed no correlation between MRI and US findings and concluded that MRI is the most appropriate imaging technique for detecting TMD in JIA patients [8, 22]. Power Doppler US was also used to assess TMJ in patients affected by JIA but its sensitivity to detect inflammation of TMJ was poor with low positive predictive value [23]. On the other side, other studies support the use of US for assessment and follow-up of TMJ involvement in patients with JIA [12, 24, 25]. Nonetheless, all these studies are limited and heterogeneous in terminology, diagnostic parameters, and study methods [26, 27].

This study aimed to evaluate whether US, compared with MRI, can be considered a suitable imaging technique to assess active TMJ disease in patients with JIA.

## Materials and methods

This study was conducted according to the ethical principles for medical research. It was approved by our local ethics committee (Protocol number 2831, October 9, 2017). All children and parents were informed about the study protocol. Informed consent was obtained from all participants.

### Sample selection

The study group (JIA group) included children diagnosed with JIA using the International League of Associations for Rheumatology (ILAR) 2001 diagnostic criteria [28]. The children with JIA presented TMJs involved by an active arthritis, as confirmed by the presence of mild or moderate/severe enhancement in CE-MRI [29]. In patients with JIA, the MRI enhancement was used as a discriminative sign of active inflammation, because effusion and bone marrow edema are always observed along with synovitis [30].

The patients were recruited at our institution by the Dental Clinic, and they were referred to our centre by the Pediatrics Clinic.

The control group (healthy group) consisted of healthy children that matched the patients with JIA for sex and age. In addition, they had healthy TMJs characterized by the absence of TMD or TMJ trauma, absence of rheumatic or systemic disease, and no pharmacological treatment. The healthy controls were recruited from the Dental Clinic.

Both groups have no previous or in progress gnathological or orthodontic treatments.

The JIA and healthy groups included the patients that were enrolled in a previous pilot study [31].

### Experimental design and procedures

In this study, the lateral periarticular space (LPAS) width in patients with JIA and active TMJ disease were measured with US. In JIA group, the US measurements of the LPAS width were compared with the ones calculated in the correspondent CE-MRI images in order to identify the level of agreement. The time interval between the two imaging methods (US and CE-MRI) was at most 1 week. In JIA group, the periarticular TMJ enhancement on CE-MRI was also assessed and classified as mild, moderate/severe.

The LPAS width of TMJs was also measured in healthy children by means of US. The healthy group was included to obtain the normal range for LPAS width in children and compared it with the LPAS values found in the JIA group.

The LPAS contains articular cavity with synovial liquid, articular disc if it is displaced, synovium and capsule. We considered the LPAS width, because the US examination is able to assess only the lateral area of the TMJ. In addition, we used the LPAS, because previous studies have demonstrated the presence of a correlation between US measurements of LPAS width and MRI findings of active inflammation such as joint effusion, synovial thickening, and joint enhancement [24, 25]. Therefore, an increase in LPAS width should be a sign of active TMJ inflammation.

US and the CE-MRI of TMJs were performed and assessed by the same expert radiologist (A.B.), with 15 years of experience in dentomaxillofacial radiology. The same radiologist performed the measurements of LPAS both on US images and on CE-MRI images.

LPAS measurements, performed both in US and CE-MRI, were repeated twice by the same radiologist in 15 randomly selected patients after a wash-out period of 1 week in order to evaluate the intraobserver agreement.

On CE-MRI images, the LPAS width and the periarticular TMJ enhancement were evaluated using the department’s picture archiving and communication system (Intellispace PACS Radiology, Philips, Amsterdam, The Netherlands).

#### MRI protocol

All MRI examinations were performed using a 1.5 T MRI scanner (Magnetom Aera, Siemens, Erlangen, Germany). MRI examinations were obtained before and after intravenous injection of a gadolinium-based contrast medium. The postcontrast MRI images were acquired on the axial plane with a T1-weighted fat saturated sequence (TR, 553 ms; TE, 12 ms; FOV, 230 mm; matrix, 218 × 448; voxel size 0.5 × 0.5 × 3mm; scan time, 2 min and 28 s).

**Fig. 1:**
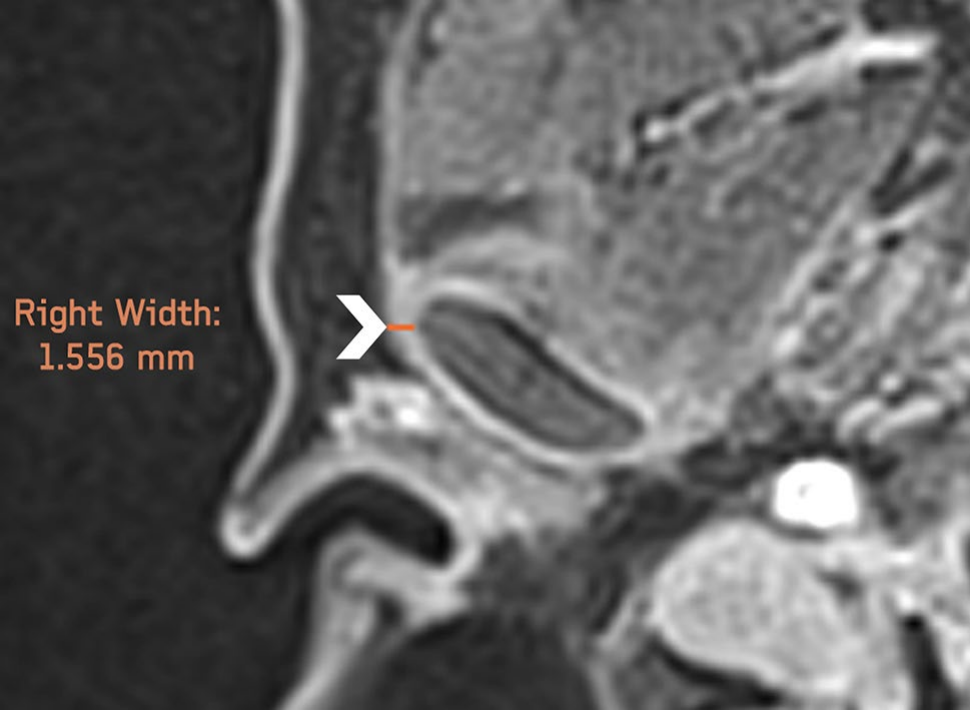
Axial postcontrast MRI image of the right temporomandibular joint. The lateral periarticular space width was measured from the most lateral point of the condylar cortex to the lateral limit of the periarticular tissue (arrowhead)

The LPAS width was assessed on postcontrast axial images at the level of the largest cross-sectional area of the mandibular condyle. The LPAS measurement was obtained using as a reference point the most lateral point of the condylar cortex until the lateral limit of the periarticular tissue (Fig. 1).

#### US protocol

All US examinations were performed with a US machine (MyLab70XVG-6150, Esaote, Genoa, Italy) using a probe with a frequency of 15 MHz.

The US images were obtained with patient in a supine position with the head turned to the left to assess the right TMJ and to the right to assess the left TMJ, in accordance with Emshoff, Jank [32]. The US probe was placed at the level of both TMJs. The LPAS was measured in a longitudinal plane parallel to the condylar neck with a close mouth position. For this study, the closed-mouth images were preferred for LPAS measurement because they were more appropriate to assess the TMJ structures due to the absence of motion artifacts [31]. For each patient, the US examination lasted about 8–10 min. During the US examination, the radiologist was blinded to clinical patients’ group origin (JIA vs healthy) symptoms/signs, and previous imaging findings.

**Fig. 2:**
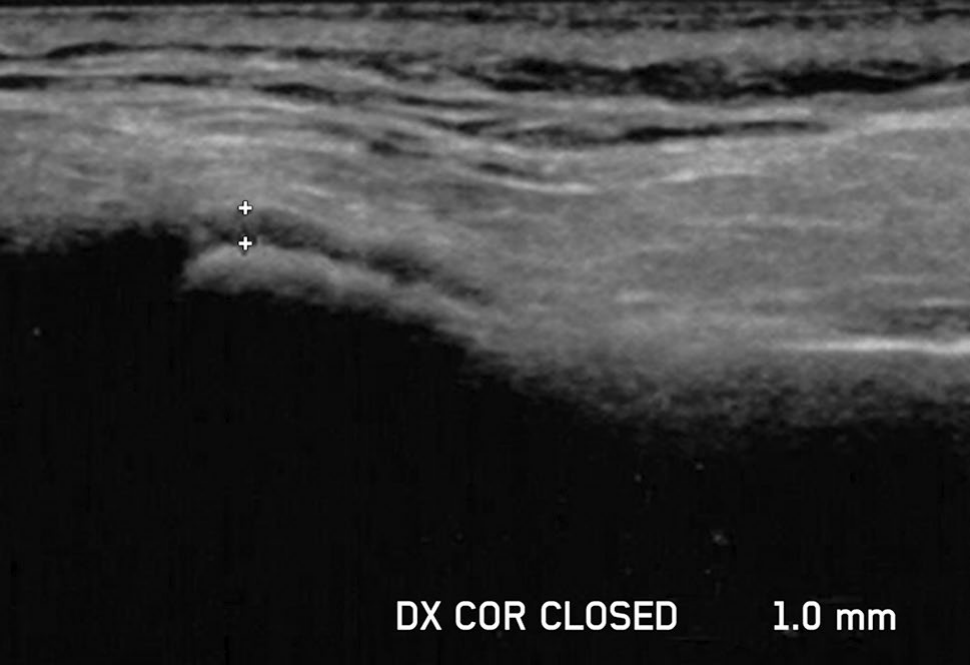
Coronal ultrasound image of the right temporomandibular joint obtained in closed-mouth position. The two reference point markers identified the hypoechoic stripe above the most lateral point of the condylar cortex, corresponding to the width of the lateral periarticular space

The width of LPAS was measured orthogonally from the lateral cortical plate of the mandibular condyle to the outline of the capsule on coronal US images [33] (Fig. 2).

### Statistical analysis

The distribution of continuous variables was tested using the Shapiro–Wilk test. Normally distributed data were presented as mean and standard deviation, whereas non-normally distributed data were presented as median and interquartile ranges (IQRs). Categorical variables were reported as relative frequencies and percentages.

The US and MRI measurements of LPAS width were not normally distributed; therefore, nonparametric tests were used. Comparisons of US and MRI measurements of LPAS between the JIA and the healthy groups were performed by applying the Mann–Whitney *U* test. In the JIA group, the Mann–Whitney *U* test was also used to verify differences of LPAS width between TMJs with mild enhancement and those with moderate or severe enhancement on CE-MRI images.

Bland–Altman method was applied to assess the agreement between two LPAS consecutive repeated measures (obtained both with US and MRI) and the agreement between MRI and US measurements of LPAS. Correlations between MRI and US values of LPAS was assessed by means of the Spearman’s correlation coefficient.

The statistically significant level was set at 5% (*p* < 0.05). STATA17 (Stata Corp., College Station, TX, USA) and MedCal (MedCalc Statistical Software version 19.2.6, Ostend, Belgium) were used to perform statistical analyses.

## Results

The JIA group included 29 JIA patients, 26 (89.7) females and 3 (10.3) males. 27/29 (93.1%) JIA patients presented bilateral TMJ active disease, whereas the remaining 2/29 (6.9%) JIA patients showed only one-sided TMJ disease. Therefore, an overall of 56 TMJs were considered in the JIA group.

The healthy group included 56 TMJs of 28 healthy children, 24 (85.7) girls and 4 (14.3%) boys. Patients’ characteristics, clinical and MRI findings at the time of the examinations are listed in Table 1.

**Table 1:** Characteristics, clinical and MRI findings of the JIA group at the time of the US examination; characteristics and clinical findings of the healthy group at the time of TMJ US examination

Variable	Values	
	JIA group	Healthy group
Age at examination (years)	13 ± 2.8	12.6 ± 2.5
Males (%)	3 (10.3)	4 (14.3)
Age at symptom onset (years)	4.8 ± 3.4	NA
Age at disease onset; doctor’s diagnosis (years)	5.4 ± 3.5	NA
Duration of the JIA disease (years)	8.4 ± 3.9	NA
Number of active joints^1^ onset	1 (1–5)	NA
Number of patients on medication (%)	24 (82.7)	NA
NSAIDs	2 (6.9)	NA
DMARDs	14 (48.3)	NA
Steroids	1 (3.4)	NA
Biological medication	14 (48.3)	NA
Other medications	4 (13.8)	NA
JIA, ILAR subtypes (%)		–
Oligoarthritis ANA negative	8 (27.6)	NA
Oligoarthritis ANA positive	15 (51.7)	NA
Polyarthritis, ANA negative	2 (6.9)	NA
Polyarthritis, ANA positive	4 (13.8)	NA
Other JIA comorbidities		NA
Crohn's disease	2 (6.9)	–
Number of patients with (%)		
TMJ pain on palpation	9 (31)	–
Muscles pain	13 (44.8)	–
TMJ sounds	14 (48.3)	–
Limited mouth opening (< 40 mm)	12 (41,4)	–
Mouth opening deviation (> 3 mm)	6 (20.7)	–
Skeletal changes^2^	9 (31)	–
Number of TMJ with MRI alterations (%)		
Joint enhancement	56 (97)	NA
Joint effusion	12 (21.4)	NA
Bone marrow edema	2 (3.6)	NA
Osteophytes	2 (3.6)	NA
Condylar flattening	23 (41.1)	NA
Erosion	16 (28.6)	NA
Disc dislocation	10 (17.9)	NA
Disc alterations	20 (35.7)	NA

Data are presented as the mean ± standard deviation, number (percentage) or median (range)

*ANA* antinuclear antibodies, *DMARDs* disease-modifying anti-rheumatic drugs, *ILAR* International League of Associations for Rheumatology classifications, *JIA* Juvenile Idiopathic Arthritis, *MRI* magnetic resonance imaging, *NSAIDs* non-steroidal anti-inflammatory drugs, *SD* standard deviation, *NA* not applicable

^1^Active joints were swollen joints or mobility restricted plus tender or painful joints

^2^Including asymmetry and retrognathia

### Comparison among groups

In the JIA group, the median width of LPAS measured on CE-MRI was 1.3 mm (IQR, 1.2–1.5 mm), whereas on US was 1.0 (IQR, 0.8–1.1 mm). In the healthy group, the median width of LPAS measured on US was 0.6 mm (IQR, 0.5–0.7 mm). Based on these findings, the LPAS width measured on US was significantly greater in the JIA group than in the healthy group (*p* < 0.0001).

In the JIA group, 15/56 (26.8%) TMJs showed moderate/severe enhancement and 41/56 (73.2%) TMJs showed mild enhancement. In TMJs with moderate/severe enhancement, the median width of LPAS measured on CE-MRI was 1.4 mm (IQR, 1.2–1.7 mm), whereas on US was 1.1 mm (IQR, 0.9–1.8 mm). In TMJs with mild enhancement, the median width of LPAS measured on CE-MRI was 1.2 mm (IQR, 1.1–1.4 mm), whereas on US was 0.9 mm (IQR, 0.8–1.1 mm). Based on these data, the LPAS width was significantly greater in TMJs with moderate/severe enhancement than those with mild enhancement (*p* ≤ 0.0133 for both US and CE-MRI).

A good level of intraobserver agreement was observed in the analysis of the two consecutive LPAS measurements performed both on MRI and on US of 15 randomly selected patients.

**Fig. 3:**
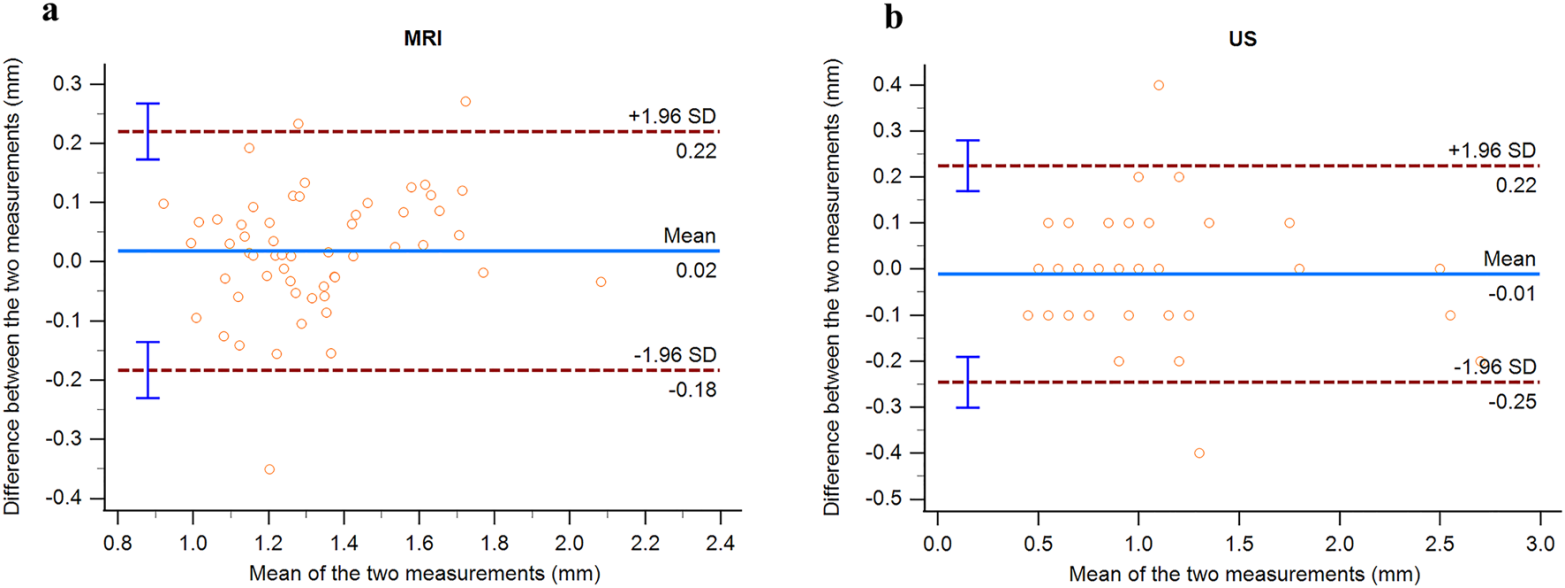
**a, b** Bland–Altman plots—measurement variability of MRI and US

The mean difference in MRI measurements was 0.02 mm (95% limit of agreement, − 0.18–0.22 mm); the 95% confidence interval (CI) of the lower limit of the agreement ranged from − 0.23 to − 0.14 mm, and the 95% CI of the upper limit of agreement ranged from 0.17 to 0.27 mm (Fig. 3a).

The mean difference in US measurements was − 0.01 mm (95% limit of agreement, − 0.25–0.22 mm); the 95% confidence interval (CI) of the lower limit of the agreement ranged from − 0.30 to − 0.19 mm, and the 95% CI of the upper limit of agreement ranged from 0.17 to 0.28 mm (Fig. 3b).

**Fig. 4:**
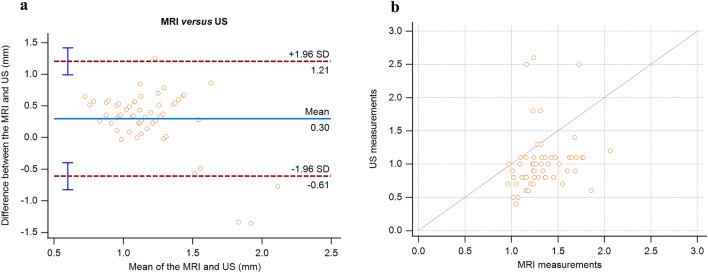
**a, b** Agreement and correlation between MRI and US measurements

Comparing LPAS measurements between MRI and US in patients with JIA, the mean difference was 0.30 mm (range − 0.62–1.21 mm); the 95% confidence interval (CI) of the lower limit of the agreement ranged from − 0.83 to − 0.40 mm, and the 95% CI of the upper limit of agreement ranged from 0.99 to 1.42 mm (Fig. 4a).

In patients with JIA, a positive significant correlation was found between MRI and US measurements (*r* = 0.403, *p* = 0.002) (Fig. 4b).

**Fig. 5:**
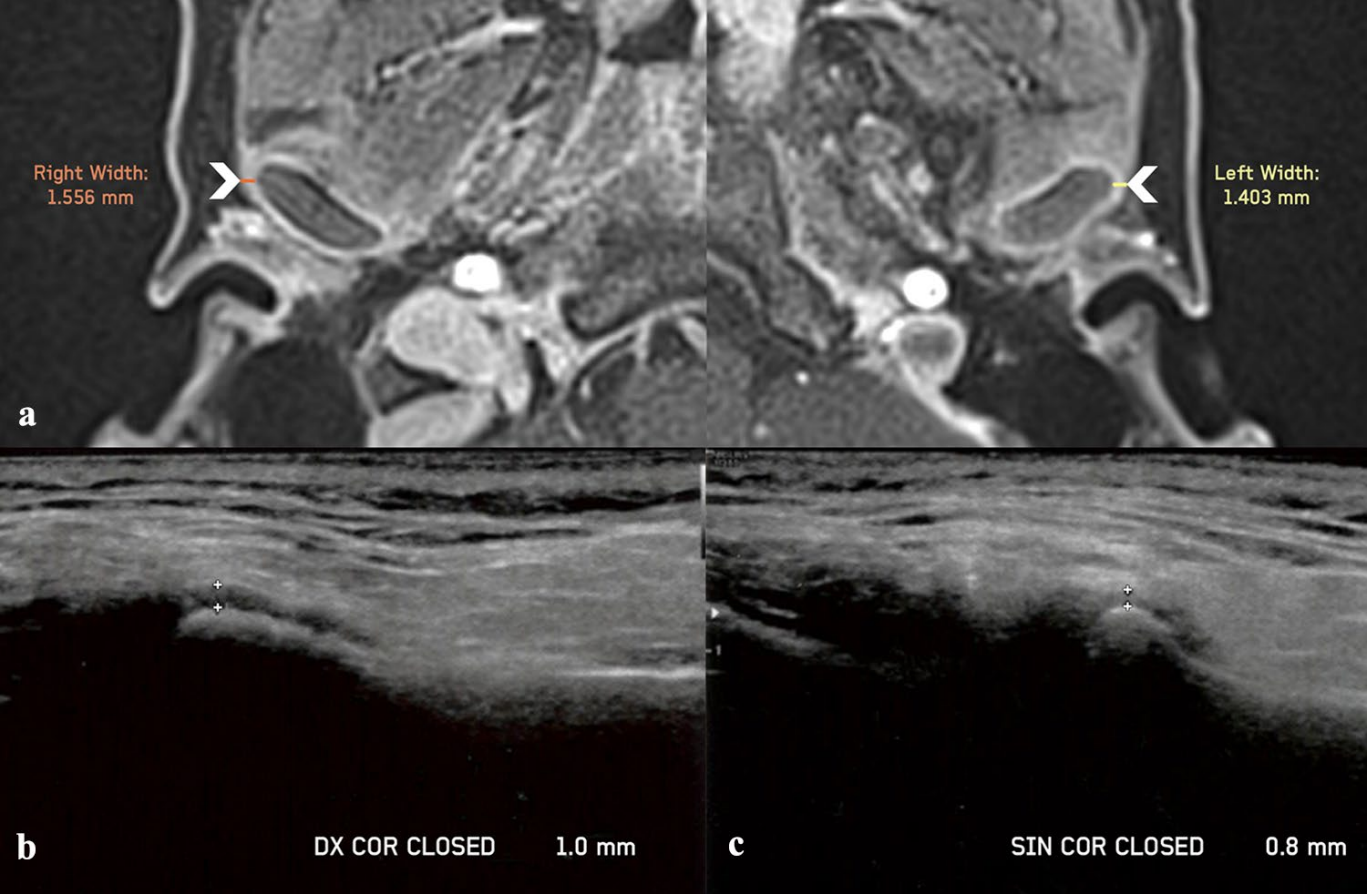
Lateral periarticular space (LPAS) assessment with MRI and US in a patient with JIA and active involvement of both TMJs. **a** Axial postcontrast MRI images of the right and the left TMJs show periarticular enhancement, with an LPAS width of 1.556 mm and 1.403 mm, respectively (arrowheads). **b**, **c** US images of the right and left TMJs show an LPAS width of 1 mm and 0.8 mm, respectively

An example of the LPAS assessment with MRI and US in a patient with JIA is shown in Fig. 5.

## Discussion

US is a non-invasive imaging method, biologically harmless, relatively fast and widely available. On the other side CE-MRI, which is the gold standard for evaluating TMJ involvement in patients with JIA, is a relatively long and not always easily accessible examination.

This study aimed to judge the possibility of using US as an alternative imaging method to assess TMJ in JIA patients, thus, it was compared with CE-MRI.

As usually observed in patients with JIA, the subjects enrolled in the present study were predominantly female. Contrary to previous studies [8, 10, 12], pain and other clinical signs of TMJ involvement (TMJ sounds, limited mouth opening, mouth opening deviation) were more frequent in our JIA patients. This is due to the fact that the children enrolled in this study were all affected by a TMJ active disease as confirmed by CE-MRI. Further, pain and the other clinical signs could be explained by an  altered muscles function considering that several children in this study presented chronic changes at the TMJ level.

This study measured and compared the same quantitative variable (LPAS width) in US and MRI. Most studies considered effusion as an essential factor for diagnosing of active TMJ arthritis in patients with JIA [8, 22, 24]. Melchiorre, Falcini [24] compared the increased thickness of the joint in US (LPAS) and joint effusion in MRI. Other studies considered only qualitative variables in US and MRI. The study of Weiss, Arabshahi [8] compared US active TMJ findings (defined as fluid collection) with MRI active TMJ findings (defined as effusions or synovial thickening). The study of Müller, Kellenberger [22] compared joint effusion and thickening of the joint capsule found in US with the presence of an effusion and increased contrast enhancement found in MRI. Nevertheless, both qualitative studies did not provide ultrasound definitions of the valued parameters. However, using effusion as discriminating factor for the diagnosis of TMJ active inflammation has some critical points to take into consideration:

- the hypoechoic area between the cortical outline of the mandibular condyle and the outline of the capsule contains articular cavity, fluid, articular disc if it is laterally displaced, synovial membrane, and these structures are not easily differentiated;
- effusion was detected with MRI in only 21,4% of the TMJs in the JIA group, despite 97% of the same TMJs presented mild/moderate or severe enhancement;
- effusion evaluated in MRI is usually seen in the anterior portion of the condyle and not in the lateral portion where the LPAS measurement is taken in US.

Power Doppler US was used to evaluate synovial vascularity in only one study [23]. However, no association was found with synovial enhancement detected by MRI. 

The US LPAS measures, which were performed in the present cohort of patients with JIA and established TMJ active arthritis by CE-MRI, showed a mean of 1.036 mm with a range of 0.6–1.5 mm. This mean value is lower compared with the periarticular width cut off value for the assessment of TMJ active inflammation used in two previous studies by Weiss, Arabshahi [8] and Müller, Kellenberger [22]. The cut off values was 2 mm and was determined for adults, which might have influenced the low correlation between periarticular width values detected by US and MRI in these two studies. Melchiorre, Falcini [24], who compared in children the increased thickness of the periarticular width in US with joint effusion in MRI, established a cut off value for the assessment of TMJ active inflammation of 1.5 mm. However, Melchiorre, Falcini [24] did not have a control group in their study. A cut off value of 1.5 mm could be too high considering that in the present study a mean LPAS width of 0.6 mm (IQR, 0.5–0.7 mm) was observed in children with healthy TMJs.

Further, the lower US LPAS mean value observed in our study could be influenced by the mandibular condylar cortical level where the LPAS measurements were performed. To reduce the changes in width due to disc displacement, Kirkhus, Gunderson [25] measured the LPAS at the subcortical level. In our work, the radiologist identified the LPAS as the hypoechoic stripe above the most lateral point of the condylar cortex [31]; it has already been demonstrated that this approach allows identifying the lateral cortical point quickly and repeating LPAS measurements with a good level of agreement [31]. This accuracy of the LPAS measurements was confirmed by the excellent level of agreement observed between two consecutive US measurements. Further the frequency of disc displacement in the JIA patients enrolled in this study was only 17.9% with no lateral displacement, whereas the frequency of disc alterations was 35.9%.

The Spearman correlation coefficient showed a positive significant correlation between MRI and US measurements of LPAS. A similar correlation was found by Kirkhus, Gunderson [25] between capsular width detected with US and presence of synovitis in MRI. The sample group in the study by Kirkhus, Gunderson [25] included JIA patients with and without TMJ synovitis allowing them to calculate a cut off value for the capsular width in US. The sample in the present study included only TMJs of patients with JIA and active involvement and it was not possible to find a cut off value for the LPAS in US. However, the LPAS width in the JIA group was significantly higher compared with the LPAS width found in the healthy group. In addition, we also found that the LPAS width (measured both on US and MRI) was significantly greater in TMJs with moderate/severe enhancement than those with mild enhancement. According to our results, the LPAS width could be used as a reference point to assess active inflammation in the TMJs of patients with JIA.

According to the literature, the present study findings suggest that US could be considered a clinically acceptable imaging modality to evaluate the TMJ involvement in patients affected by JIA [12, 24, 25]. In the case of non-cooperative patients, such as children, patients with claustrophobia and when the MRI exam is not available, the measure of the LPAS in US could help in the evaluation of TMJ involvement in patients with JIA as already shown by Assaf, Kahl-Nieke [12]. The LPAS evaluation with US could also be used as a screening method, as already claimed by Melchiorre, Falcini [24] and Kirkhus, Gunderson [25].

In children without clinical signs and symptoms of TMJ involvement or in the presence of unclear cases after the evaluation of the five clinical variables used by Stoustrup, Herlin [16] as indicators of possible TMJ involvement (TMJ pain on palpation, muscles pain on palpation, mandibular deviation ≥ 3 mm, reduced maximal mouth opening, frontal facial asymmetry and deformity of the facial profile), the US may be a valuable imaging modality in the decision to additional MRI investigation. Finally, US could also be helpful in the follow-up of the disease progression at the TMJ level [12, 24]. After the diagnosis of TMJ active or chronic involvement has been made by the MRI examination; the temporomandibular joint could be monitored using US. Successive US investigations could be carried out to assess the decrease or increase in the size of the LPAS, which would indicate improvement/rest or worsening/reactivation of the arthritic involvement of the TMJ.

The strength of this study is that it is a prospective study with a control group. The JIA patients undertook the two imaging techniques (US and CE-MRI) at a maximum of 1 week apart, the same US and MRI scanners were used for all the patients and the same expert radiologist evaluated both the MRI and US images.

Our study presents some limitations. First, the sample size was small and patients were referred by the Pediatrics Clinic of only one hospital. Thus, some categories of JIA were not present, and oligoarthritic and polyarthritis were more frequent than in epidemiological studies [34], respectively, 80% and 20%. Second, ultrasonography is a strongly operator-dependent imaging technique. Third, only intraobserver agreement analysis was evaluated in this study, because the US scans were performed by only one experienced observer. Therefore, future prospective multi-centre studies, with larger sample sizes, and more than one observer should be conducted to further confirm the promising role of US in assessing the TMJ disease in patients with JIA.

## Conclusions

Currently, US cannot replace MRI but it could be used as a supplementary imaging method to assess temporomandibular joint involvement in JIA patients; especially in those cases when MRI is not available or children are not cooperative, and for the initial screening of TMJ involvement or the follow-up of the disease progression to avoid repeated MRI checks and related costs.
